# Cellular Reprogramming Employing Recombinant Sox2 Protein

**DOI:** 10.1155/2012/549846

**Published:** 2012-05-29

**Authors:** Marc Thier, Bernhard Münst, Stephanie Mielke, Frank Edenhofer

**Affiliations:** Stem Cell Engineering Group, Institute of Reconstructive Neurobiology, University of Bonn-Life & Brain Center and Hertie Foundation, Sigmund-Freud Straße 25, D-53105 Bonn, Germany

## Abstract

Induced pluripotent stem (iPS) cells represent an attractive option for the derivation of patient-specific pluripotent cells for cell replacement therapies as well as disease modeling. To become clinically meaningful, safe iPS cells need to be generated exhibiting no permanent genetic modifications that are caused by viral integrations of the reprogramming transgenes. Recently, various experimental strategies have been applied to accomplish transgene-free derivation of iPS cells, including the use of nonintegrating viruses, episomal expression, or excision of transgenes after reprogramming by site-specific recombinases or transposases. A straightforward approach to induce reprogramming factors is the direct delivery of either synthetic mRNA or biologically active proteins. We previously reported the generation of cell-permeant versions of Oct4 (Oct4-TAT) and Sox2 (Sox2-TAT) proteins and showed that Oct4-TAT is reprogramming-competent, that is, it can substitute for Oct4-encoding virus. Here, we explore conditions for enhanced Sox2-TAT protein stabilization and functional delivery into somatic cells. We show that cell-permeant Sox2 protein can be stabilized by lipid-rich albumin supplements in serum replacement or low-serum-supplemented media. Employing optimized conditions for protein delivery, we demonstrate that Sox2-TAT protein is able to substitute for viral Sox2. Sox2-piPS cells express pluripotency-associated markers and differentiate into all three germ layers.

## 1. Introduction

Pluripotent cells represent a most attractive source for both cell repair in regenerative medicine and disease modeling in basic biomedical research since they are able to differentiate into every cell type of an adult organism. Until recently, early embryonic stages of development represented the main source of pluripotent cells, and thus, those cells were designated as embryonic stem (ES) cells. Nowadays, the artificial derivation of pluripotent stem cells from somatic cells becomes increasingly important. Induced pluripotent stem (iPS) cells were first generated by retrovirally induced ectopic expression of four transcription factors Oct4, Sox2, Klf-4, and c-Myc in somatic cells [1]. Human iPS cells represent an attractive option for the derivation of pluripotent patient-specific cells as no embryos are required for their generation. However, crucial safety issues have to be addressed in order to generate human iPS cells that are clinically useful. Soon after identification of the viral reprogramming protocol in mouse cells [1] and its adaptation to human cells [2, 3], unwanted side effects such as tumorigenesis [4] became apparent.

Since the cause of tumor formation was ascribed to random integration of the retroviral vectors and sustained expression of transgenes after reprogramming, optimized protocols were explored to circumvent the permanent integration of foreign DNA into the genome. One strategy involves the excision of reprogramming transgenes employing DNA recombinases [5, 6] or transposases [7–10]. After iPS derivation, transgenes can be deleted by a second round of recombinase/transposase activation. However, further laborious and cumbersome genetic methods are needed to identify and confirm transgene-free iPS clones. An alternative strategy is to utilize less-invasive genetic vectors that do not integrate into the host genome. Repeated plasmid transfection has been used for iPS induction albeit with a very low efficiency [11]. Minicircle vectors lacking bacterial DNA and thus enabling high transfection efficiency and long ectopic expression were reported to reprogram as well [12]. Moreover, transduction employing viruses that do not integrate their genome into host cells such as adenovirus [13] or Sendai virus [14] were applied. Small molecules that are able to translocate into cells and interfere with key signaling pathways have been identified to either enhance the process of reprogramming [15, 16] or replace [15, 17] single viral factors (for review, see [18]). The repeated transfection of synthetic mRNA [19–21] or the direct delivery of reprogramming proteins [22, 23] represents a straightforward but technically challenging approach to achieve nongenetic iPS derivation.

Protein transduction technology has been used to directly deliver numerous biologically active proteins into mammalian cells by modifying them with so-called cell-penetrating peptides (CPPs) or protein transduction domains (PTDs). These relatively small peptides confer cell permeability when linked to cargo molecules (for review, see [24–26]). A highly basic CPP derived from the *human immunodeficiency virus type 1* (HIV-1) Tat (transactivator of transcription) protein is often applied for cellular delivery (TAT) [25–28]. PTDs have been used to generate cell-penetrating versions of various transcription factors that play major roles in cell differentiation including HoxB4 [27], Pdx1 [28], Scl [29], Nkx2.2 [30], and Notch-ICD [31]. We previously reported the derivation of cell-permeant versions of reprogramming factors Oct4 and Sox2 [22]. Oct4-TAT and Sox2-TAT were shown to specifically bind to DNA such as the Oct4/Sox2 combined element in the Nanog promoter, and both proteins compensate the RNAi-induced loss of function after direct delivery into ES cells [22]. Moreover, employing Sox2-TAT, it has been demonstrated that Sox2 has an essential function in the preimplantation mouse embryo by facilitating establishment of the trophectoderm lineage [32]. Zhou et al. used fusion protein derivatives of reprogramming factors from *E. coli* for the derivation of mouse ES-like cells, albeit with very low efficiency [23]. Kim et al. reported the use of cell extracts from transfected HEK293 cells for the reprogramming of human newborn fibroblasts [33]. The recently reported use of ES cell extracts to induce pluripotency in murine fibroblasts [34] needs to be explored whether it can be adapted to human cells. In conclusion, a robust, standardized, and efficient protocol for the generation of protein-induced iPS cells from human adult cells still needs to be developed.

Further optimization of protein transduction for cellular reprogramming greatly depends on overcoming a major bottleneck associated with protein transduction: stability of recombinant factors under cell culture conditions. We recently established optimized stabilization conditions for Oct4-TAT and demonstrated the efficient substitution for Oct4-encoding virus by recombinant Oct4-TAT [36]. Here, we explore conditions for enhanced Sox2-TAT protein stabilization and delivery into somatic cells. We show that cell-permeant Sox2 protein can be stabilized by lipid-rich albumin supplements in serum replacement or low-serum-supplemented media. Employing these conditions for protein delivery, we demonstrate that Sox2-TAT protein is able to substitute for viral Sox2.

## 2. Materials and Methods

### 2.1. Protein Expression and Purification

The pSESAME-Sox2NTH expression plasmid [22] was transformed into *E. coli *BL21 (DE3) gold strain (Stratagene, La Jolla, USA) by heat shock at 30°C and incubated for 1 h in SOC medium at 30°C. Transformed bacteria were inoculated overnight at 30°C with shaking at 140 rpm in LB medium containing 50 mg/mL carbenicillin. For protein expression, the overnight culture was pelletized and resuspended in fresh TB medium (terrific broth)/50 mg/mL ampicillin, 0.5% glucose and incubated at 37°C with shaking at 110 rpm until an OD600 of 1.5 was achieved. Protein expression was induced by IPTG at a final concentration of 0.5 mM. Cells were harvested by centrifugation, and cell pellets were stored at −20°C.

For purification of His-tagged proteins, cell pellets were thawed and resuspended in 20 mL lysis buffer (50 mM Na_2_HPO_4_, 5 mM Tris, pH 7.8, 500 mM NaCl, and 10 mM imidazole) per 1 L of expression culture. Cells were lysed by application of 1 mg/mL lysozyme (Sigma, Deisenhofen, Germany), 10–15 U/mL Benzonase (Novagen, Darmstadt, Germany), and sonication. After centrifugation (17 200 g, 20 min), the cleared lysate was incubated with Ni-NTA agarose beads (Qiagen, Hilden, Germany) (1 mL of slurry for 1 L of bacterial expression culture) for 1 h with rotation at 4°C. The slurry was packed into a column and washed with 8 column volumes of wash buffer (50 mM Na_2_HPO_4_, 5 mM Tris, pH 7.8, 500 mM NaCl, and 80 mM imidazole). The Sox2-TAT protein was eluted with 3 column volumes of elution buffer (50 mM Na_2_HPO_4_, 5 mM Tris, pH 7.8, 500 mM NaCl, and 250 mM imidazole).

### 2.2. Preparation of Transduction Media

Sox2-TAT eluate fraction was supplemented with 7.5% serum replacement and dialyzed against DMEM F12 over night at 4°C. The next day cell culture supplements were added to the dialyzed fraction to a final concentration of 2% FCS, 2.5% Albumax II (200 mg/mL), 7.5% serum replacement, 1% ITS, 0.1 mM nonessential amino acids, 1 mM sodium pyruvate, 2 mM L-glutamine, 0.5 mM *β*-mercaptoethanol, and 1000 U/mL LIF. The mixture was preconditioned in a water bath for 1 h at 37°C and cleared by centrifugation (5 min at 2500 g) and sterile filtration.

### 2.3. Cell Culture

Oct4-GiP MEFs [38] were cultured in high-glucose DMEM (Invitrogen) with 10% FCS, 0.1 mM nonessential amino acids, 1 mM sodium pyruvate, and 2 mM L-glutamine. MEFs were trypsinized at 70–80% confluence and reseeded on tissues culture dishes coated with gelatin. For reprogramming assays, MEFs were used to a maximum of passage 4.

mESCs were cultured in DMEM F12 (Invitrogen) supplemented with 2% FCS, 2.5% Albumax II (200 mg/mL), 7.5% serum replacement, 1% ITS, 0.1 mM nonessential amino acids, 1 mM sodium pyruvate, 2 mM L-glutamine, 0.5 mM *β*-mercaptoethanol, and 1000 U/mL LIF. Cells were split every 3 days and cultured on inactivated feeder cells.

### 2.4. Retroviral Infection and iPS Induction

 Plasmids of pMXs-Oct3/4, pMXs-Sox2 (positive control), pMXs-c-Myc and pMXs-Klf4 were obtained from ADDGENE. The retroviruses were generated by the plat E packaging cell line as previously described [39]. Target cells were seeded at 10 × 10^4^ cells per well in six-well plates. 24 hours after transfection, the supernatant comprising the viruses was collected and filtered through a 0.45 *μ*m cellulose acetate filter. For substitution experiments and for negative controls, Oct4, Klf4, and c-Myc were mixed in equal shares and supplemented with polybrene (Millipore) to a final concentration of 4 *μ*g/mL. Positive controls additionally contained pMXs-Sox2 virus and were treated alike. Oct4-GiP MEF cells were incubated with viruses for 16 hours. Protein transduction experiments began after the virus-containing supernatant had been removed. After 5 days, cells were split onto irradiated feeder cells. 11 days later, cells were fixed with 4% PFA and analyzed by fluorescence microscopy. For the purpose of generating stable iPS cell lines, reprogramming assays were cultured for 21 days under designated conditions. Subsequently, colonies were picked and expanded monoclonally.

### 2.5. In Vitro Differentiation

Cells were harvested by trypsinization and transferred to bacterial culture dishes. Next, cells were grown in ES medium lacking LIF for 3 days. The derived embryoid bodies were transferred to gelatine-coated tissue dishes afterwards and incubated for another 3 days. In order to detect key marker expression specific for all three germ layers, immunostainings with antibodies against *β*-3-tubulin (TUJ1), smooth muscle actin (SMA), and *α*-fetoprotein (AFP) were conducted. 

### 2.6. RT-PCR

Total RNA was extracted using the RNAeasy kit (Qiagen, Hilden, Germany) following the manufacturer's instructions. Subsequently, reverse transcription of 1 *μ*g RNA per sample was performed using the iScript cDNA Synthesis Kit (Bio-Rad). In order to detect viral transgene expression, the following primer pairs were used:

Oct4TGforw: CCCCACTTCACCACACTCTAC,
Oct4TGrev: TTTATCGTCGACCACTGTGC,
Klf4TGforw: AGGCACTACCGCAAACACAC,
Klf4TGrev: TTTATCGTCGACCACTGTGC,
Sox2TGforw: GCCCAGTAGACTGCACATGG,
Sox2TGrev: CCCCCTTTTTCTGGAGACTA,
c-MycTGforw: CAGAGGAGGAACGAGCTGAAGCGC,
c-MycTGrev: TTTGTACAAGAAAGCTGGGT.

For the detection of endogenous Oct4, Sox2, and Nanog, the following primer pairs were used:

Oct4forw: TCTTTCCACCAGGCCCCCGGCTC,
Oct4rev: TGCGGGCGGACATGGGGAGATCC,
Sox2for: TAGAGCTAGACTCCGGGCGATGA,
Sox2rev: TTGCCTTAAACAAGACCACGAAA,
Nanogfor: CAGGTGTTTGAGGGTAGCTC,
Nanogrev: CGGTTCATCATGGTACAGTC.

PCR-program: 95°C 2 min, 95°C 30 sec, 60°C 30 sec, 72°C 1 min, 72°C 10 min. Steps 2–4 are repeated 35 times.

## 3. Results

### 3.1. Purification of Reprogramming-Competent Sox2 Fusion Protein from Bacteria

**Figure 1:**
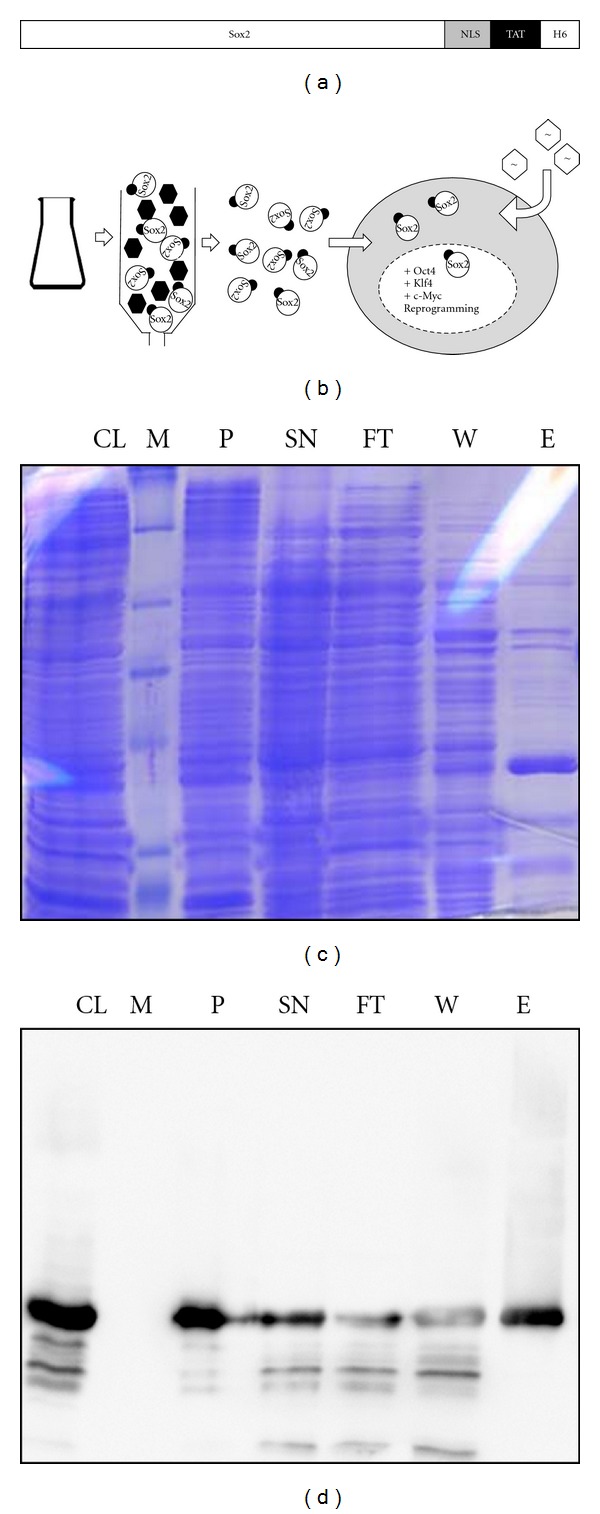
Purification of recombinant Sox2-TAT fusion protein and reprogramming setup. (a) The recombinant cell-permeant Sox2 fusion protein [22] consists of the full-length Sox2 protein and a carboxy-terminally fused sequence of tags consisting of a nuclear localization sequence (NLS), cell-penetrating peptide TAT, and a histidine-tag (H6) for single-step purification. (b) Schematic representation of the expression and purification procedure and the reprogramming setup used in this study. After expression in *E. coli*, Sox2-TAT-containing cell lysates are subjected to affinity column chromatography employing Ni-NTA resin. Purified recombinant Sox2-TAT protein is eluted from the column and its reprogramming competency assessed in combination with retroviruses encoding Oct4, Klf-4, and c-Myc. (c, d) Biochemical analysis of Sox2-TAT purification from *E. coli*. The following fractions were subjected to SDS-PAGE analysis: crude lysate (CL), marker (M), pellet (P), supernatant (SN), flow-through (FT), washing buffer (W), and elution fraction (E). SDS gels were either stained using Coomassie (c) or used for preparation of an immunoblot using anti-Sox2-specific antibody (d).

We have previously shown that recombinant Sox2 can be purified from *E. coli* as a TAT-modified cell-permeant version, designated Sox2-TAT [22]. In particular, this fusion protein comprises an additional exogenous nuclear localization sequence (NLS), a cell-penetrating peptide TAT, and a carboxy-terminal Histidine-tag for single-step purification (Figure 1(a)). Sox2-TAT was shown to specifically bind to DNA and to compensate for the RNAi-induced loss of activity in ES cells [22] and preimplantation embryos [32]; however, its capability to reprogram somatic cells has not been assessed. In order to study the reprogramming activity of Sox2-TAT, we decided to combine the purified recombinant Sox2-TAT together with retroviruses encoding for Oct4, Klf4, and c-Myc to convert mouse embryonic fibroblasts (MEFs) into iPS cells (Figure 1(b)). Sox2-TAT-transformed bacteria were lysed and subjected to Ni-affinity chromatography. Immunoblotting of purification fractions employing a His-specific antibody revealed that Sox2-TAT is highly expressed in bacteria although the majority of the recombinant protein remains in the insoluble fraction (Figure 1(d)). However, the estimated 20% of protein solubilized and detectable in the supernatant turned out to be sufficient for further purification. The elution from the Ni-affinity chromatography column yielded a Sox2-TAT-containing fraction of about 70% purity (Figure 1(c)).

### 3.2. Defining Optimal Conditions to Stabilize Sox2-TAT Protein

**Figure 2:**
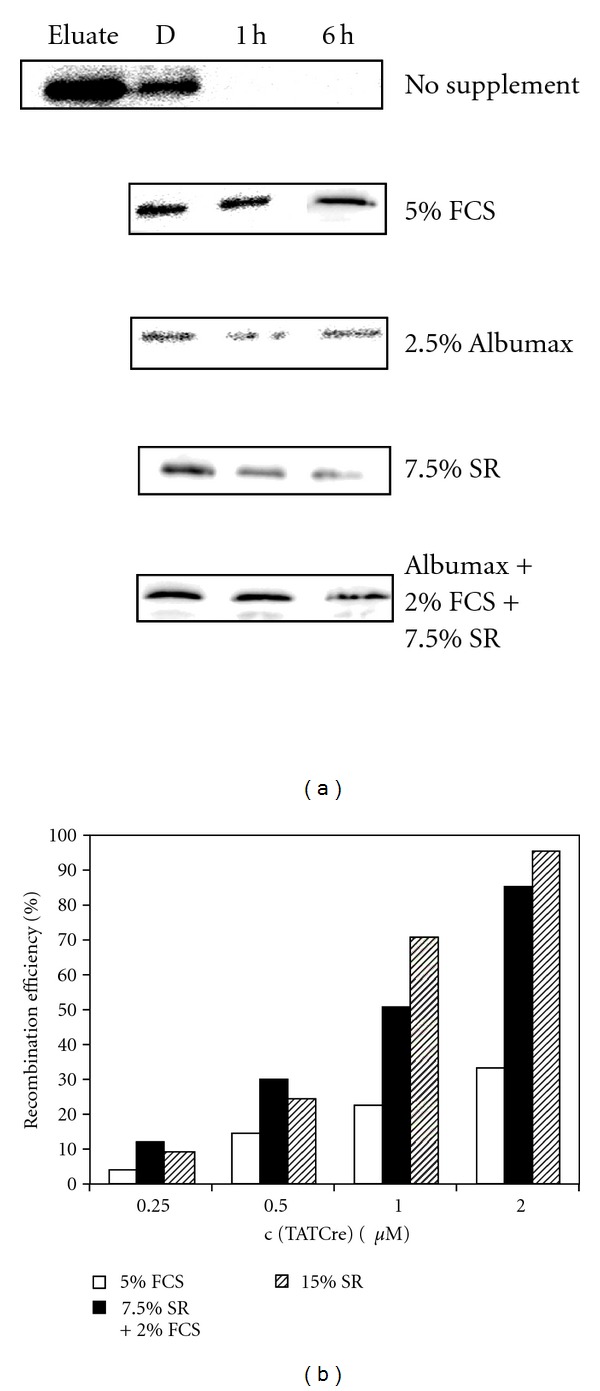
Effect of media supplements on the stability of cell-permeant Sox2-TAT fusion protein and efficiency of protein delivery. (a) Ability of media supplements to stabilize Sox2-TAT. Fetal calf serum (FCS), Albumax, and serum replacement (SR) were added to the eluate fraction and subsequently dialyzed against DMEM-F12 media. Depicted are anti-Sox2-immunoblots of the dialysis fraction (D) and a stability test of samples being taken after 1 hour (1 h) and 6 hours (6 h), respectively. (b) Influence of FCS, SR, and a combination of both on the transduction efficiency. Protein transduction efficiencies were analyzed by quantifying the recombined cells after delivery of cell permeant Cre-protein (TAT-Cre) into the CV1-5B Cre reporter cell line. Cells were treated with different concentrations of TAT-Cre (0.25 *μ*M–2 *μ*M) in transduction media supplemented with either 15% serum replacement, 5% FCS, or mixture of 2% FCS and 7.5% SR. To determine the recombination activity, cells were fixed and stained for *β*-galactosidase activity after 48 hours. Cre protein transduction and quantification of recombination in Cre reporter cells was performed as described previously [35].

Poor solubility and limited stability of recombinant proteins under cell culture conditions represent a significant hurdle to the application of protein transduction technology. On the one hand, serum components stabilize recombinant proteins in cell culture media, but on the other hand, they are known to inhibit interaction of transducible proteins with cells and by this decrease the cellular uptake. In some experimental settings, this can be overcome by applying the transducible protein in serum-free media. We assessed the stability of Sox2-TAT employing various cell culture conditions. In serum-free media, Sox2-TAT precipitates almost completely within 1 hour (Figure 2(a)). Serum components have been shown to execute a positive effect on the stability of recombinant proteins [36, 40]. Therefore, we aimed at stabilizing the protein by supplements like FCS, serum replacement [35], and lipid-rich albumin fractions (Albumax). Supplementation with either 5% FCS or 2.5% Albumax showed a strong stabilizing effect on Sox2-TAT in culture media, while Sox2-TAT exhibited major decrease in the presence of 7.5% SR. A combination of low FCS (2%) together with 7.5% SR resulted in a stabilization that was comparable to high FCS supplementation (5%) (Figure 2(a)).

Next, we set out to analyze to which extent the stabilizing supplements interfere with the protein transduction process. To that aim, we used a well-established transduction read-out system based on a cell-permeant version of the DNA recombinase Cre [40]. A major feature of this protein transduction system is that the efficiency of intracellular protein delivery can reliably be quantified by a Cre recombinase reporter assay. We used the CV1-5B Cre reporter cell line [37] that specifically expresses *β*-galactosidase after Cre-mediated recombination. Using this read-out system, we tested the influence of FCS and SR on the transduction efficiency (Figure 2(b)). 2 *μ*M of TAT-Cre in 5% FCS-supplemented media induced recombination in approximately 35% of cells, whereas more than 90% of cellular targets were recombined in medium containing 15% SR. Application of 1 *μ*M of TAT-Cre revealed a similar correlation: about 20% recombination in the presence of FCS and 70% with SR (Figure 2(b)). These data demonstrate that FCS in contrast to SR exhibits a strong inhibition on protein transduction. Based on these results, we established an optimized transduction protocol for iPS derivation employing Sox2-TAT. We decided to apply a two-step protocol to optimize both, protein stability and transduction capacity. In a first step, the eluate fraction was supplemented with 7.5% SR and dialyzed against DMEM/F12. Afterwards, the dialysis fraction was supplemented with FCS (2%) and Albumax (2.5%). The optimized medium showed protein stabilizing capacity during the dialysis and under cell culture conditions in the same range as compared to SR and FCS, respectively.

### 3.3. Reprogramming OKC-Infected Cells with Cell-Permeant Sox2 Fusion Protein

**Figure 3:**
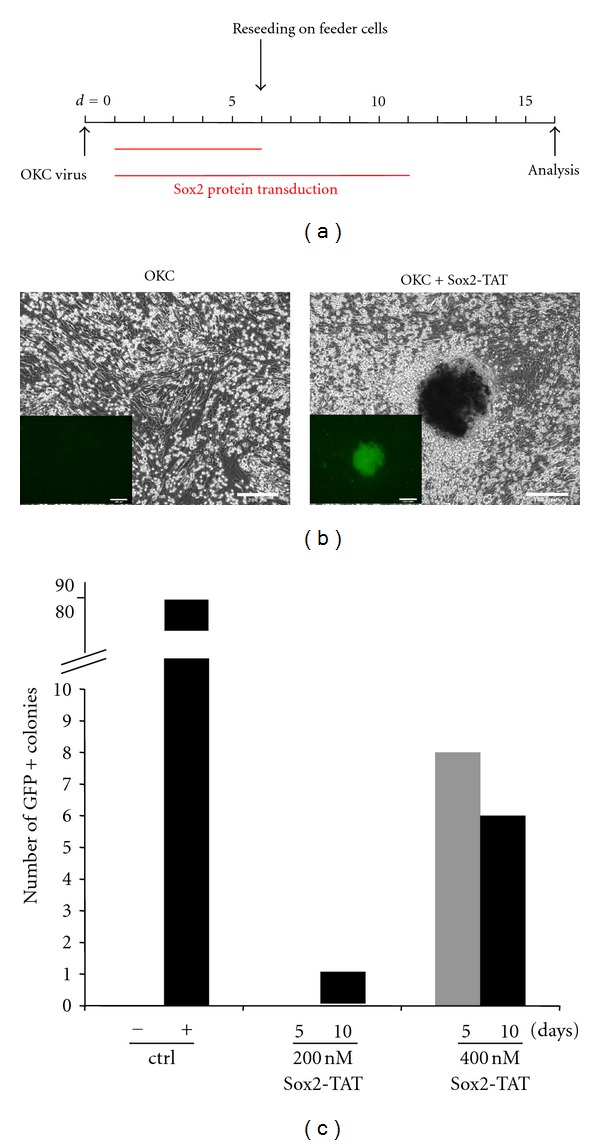
Reprogramming of MEFs using cell-permeant Sox2-TAT protein. (a) Schematic presentation showing the timeline of reprogramming setup. Oct4-GiP MEFs were infected with viruses encoding Oct4, Klf4, and c-Myc (OKC) at day 0. Starting at day 1 post infection (p.i.), cells were incubated with Sox2-TAT for 5 and 10 days, respectively, changing the Sox2-TAT-supplemented media daily. (b) Representative pictures of cells transduced with 200 nM Sox2-TAT protein (right panel) displaying phase contrast and GFP channel (inset) 14 days p.i. OKC-infected cells treated with medium only served as controls (left panel). Scale bar = 100 *μ*m. (c) Quantification of GFP-positive colonies at day 16 p.i. OKC-infected cells (−) as well as SOKC-infected cells (+) served as controls.

We then assessed the reprogramming activity of Sox2-TAT employing the optimized media conditions. For that, we used a modification of the classical four-factor viral reprogramming paradigm and aimed at substituting the Sox2-encoding virus by Sox2-TAT protein (Figure 3(a)). We transfected Oct4-GiP-transgenic MEFs [38], which enable GFP-based monitoring of reprogramming by reactivation of the Oct4 promoter, with viruses encoding for Oct-4, Klf4, and c-Myc (OKC). OKC-MEFs were initially cultivated for five days in medium containing either 200 nM or 400 nM Sox2-TAT. During the whole reprogramming procedure, we changed the protein-supplemented media every day to ensure a continuous delivery of the recombinant reprogramming factor. At day five, cells were split onto freshly plated feeder cells and either cultured in normal media or further exposed to Sox2-TAT-containing media for five more days (Figure 3(a)). First, iPS-like structures appeared after 9 days and formed well-defined GFP-positive colonies (Figure 3(b)). The viral transduction of the three factors Oct4, Klf4, and c-Myc without Sox2-TAT protein did not yield any GFP-positive colony (Figure 3(b)). GFP^+^ colonies were quantified at day 16. We counted eight GFP^+^ colonies in wells containing cells treated with 400 nM Sox2-TAT for five days. Prolonged incubation with Sox2-TAT until day 10 did not result in a marked increase of colony numbers. Instead, the number of colonies slightly decreased eventually due a strict time window required for Sox2 application. The application of 200 nM Sox2-TAT for five and ten days yielded no and just one GFP^+^ colony, respectively (Figure 3(c)), indicating that the Sox2-TAT concentration is a limiting factor. We aimed at further increasing the Sox2-TAT concentration by either dialysis against glycerol-containing concentration buffer or ultrafiltration; however, beyond 400 nM, the protein strongly precipitated in culture media and interfering with cellular growth (data not shown).

### 3.4. Sox2-piPS Cells Exhibit Pluripotency

**Figure 4:**
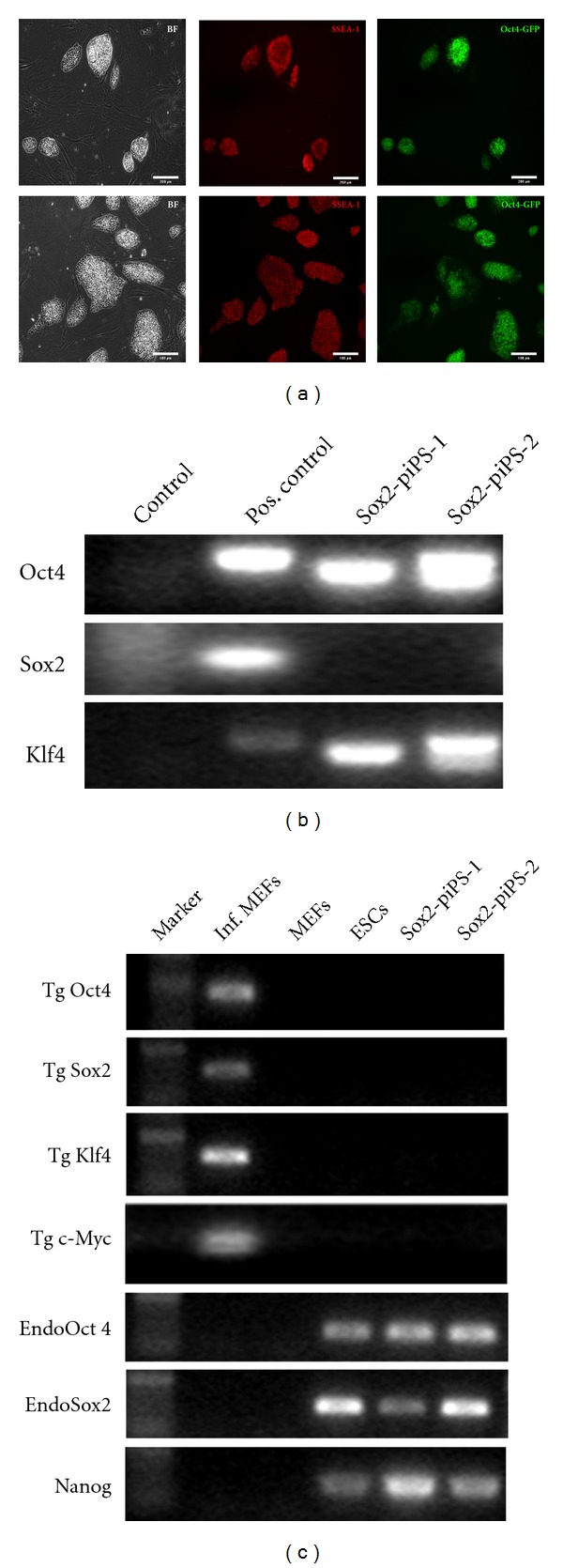
Cellular and molecular characterization of iPS clones derived by Sox2 protein transduction into OKC-MEFs. (a) Pictures of isolated cell lines Sox2-piPS-1 (upper row) and Sox2-piPS-2 (lower row) exhibiting brightfield (BF), staining against pluripotency-associated marker SSEA-1 and native GFP fluorescence. Sox2-piPS-1 cell line was clonally isolated from 400 nM Sox2-TAT treatment from day 1 to 5, and Sox2-piPS-2 was derived from 200 nM condition (day 5 to 10) Scale bar = 100 *μ*m. (b) PCR analysis of genomic DNA demonstrating genomic integration of Oct4 and Klf4 transgenes. As expected, no transgenic Sox2 was detected in Sox2-piPS clones excluding possibility of contamination. (c) RT-PCR analysis demonstrating transgene silencing in Sox2-piPS cells. Primers specific for transgenic Oct4, Sox2, Klf4, and c-Myc were used. Additionally, we analyzed endogenous Oct4, Sox2, and Nanog transcripts. RNA preparations from infected (Inf.) and uninfected MEFs as well as ES cells served as controls.

**Figure 5:**
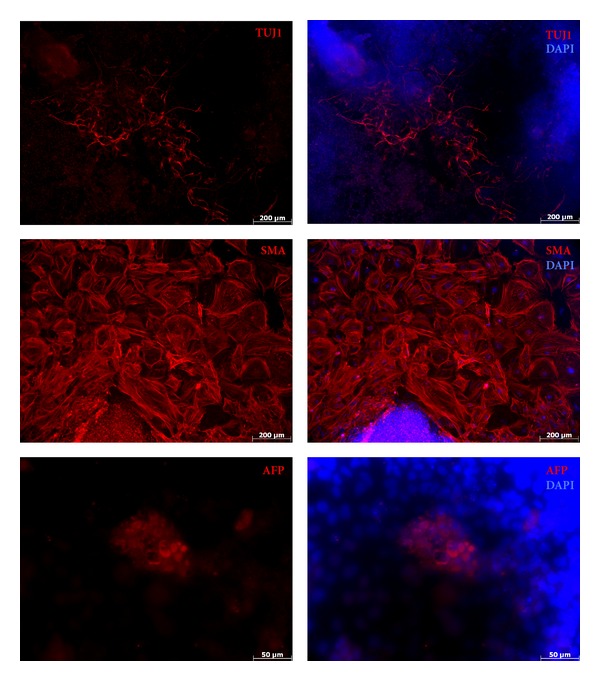
*In vitro* differentiation of Sox2-piPS cells. Spontaneously differentiated Sox2-piPS cells were stained for **β**-3-tubulin (TUJ1, ectoderm) smooth muscle actin (SMA, Mesoderm), and *α*-fetoprotein (AFP, endoderm) as indicated. DAPI co-staining was performed in every condition.

Two Sox2-protein iPS colonies were isolated and expanded for further characterization, yielding Sox2-piPS-1 and Sox2-piPS-2 cell lines, respectively. Both could be proliferated for at least 20 passages, and they maintained their Oct4 promoter-driven GFP activity. Moreover, they stained positively for the pluripotency-associated cell surface marker SSEA-1 (Figure 4(a)). Sox2-piPS-1 and Sox2-piPS-2 were subjected to PCR analysis in order to assess transgenic integrations. This analysis revealed that both lines carry integrated viral transgenes, but no exogenous Sox2 (Figure 4(b)). Transgene silencing represents a major hallmark of successful iPS derivation. Thus, we applied RT-PCR analysis to detect the transcripts of the transgenic reprogramming factors as well as endogenous stemness factors. Both clones analyzed exhibit no detectable transgenic Oct4, Sox2, Klf4, or c-Myc (Figure 4(c)). The mRNA of endogenous Oct4, Sox2 and Nanog, in contrast, was found as abundant as in the ES control cells, indicating complete silencing of exogenous factors and reactivation of the endogenous grid of stemness. Finally, we set out to confirm the pluripotent status of Sox2-piPS-2 by spontaneous differentiation into embryoid bodies (EBs). 5-day old EBs were plated and analyzed for the appearance of specific germ layer marker by staining against *β*-3-tubulin (TUJ1), smooth muscle actin (SMA), and *α*-feto-protein (AFP) (Figure 5). According to this analysis, Sox2-piPS-2 cells differentiated into all three germ layers, demonstrating an unrestricted *in vitro* differentiation potential.

## 4. Discussion

In this study, we elaborated an optimized protocol for the delivery of cell-permeant Sox2-TAT protein into mammalian cells. Poor stability of Sox2-TAT under cell culture conditions represents a major bottleneck of Sox2 protein transduction. Media supplements Albumax, SR, and FCS were analyzed for their stabilizing effect on Sox2-TAT. FCS was found to stabilize Sox2-TAT, whereas the recombinant protein rapidly precipitates in protein-free media. We used the Cre protein transduction system [40] to assess the effects of media supplements SR, FCS, and Albumax on protein transduction in a quantitative manner. It turned out that SR has no deleterious effect on cellular delivery, whereas FCS strongly reduces the cellular uptake. Thus, in terms of protein delivery, supplementation of SR is preferred over FCS. However, since in contrast to ES and iPS cells fibroblast cells do not tolerate SR we employed a mixture of 7.5% SR and 2% FCS. These media conditions represent an optimal compromise for cultivation during protein-induced reprogramming of fibroblasts.

Employing these optimized conditions, we demonstrate that Sox2-TAT is able to substitute for a Sox2-encoding virus during the OKC-viral induction of pluripotency in fibroblast cells. Stable Sox2-piPS cell lines could be generated exhibiting major hallmarks of ES cells such as pluripotency-associated marker expression and full differentiation potential *in vitro*. We found that proliferation of Sox2-piPS cells does not depend on the continuous expression of the OKC transgenes as judged by RT-PCR analysis. Notably, the transduction of 200 nM Sox2-TAT from day 5 to day 10 resulted in the induction of one GFP^+^ colony (data not shown), whereas treatment from day 1 to 5 did not give rise to any colony, albeit the same concentration of protein was used. Whether this observation hints at a specific time dependence of Sox2 during the reprogramming process or is a result from stochastic variation remains to be investigated. In general, the efficiency of Sox2-piPS derivation is at least one order of magnitude lower as compared to our previously reported generation of Oct4-piPS cells [36]. From this observation, we conclude that although we provide proof-of-principle data, that recombinant Sox2-TAT is reprogramming-competent, sufficient delivery of biologically active Sox2 protein into the right cellular compartment represents a bottleneck for protein-induced iPS derivation.

Further investigations are needed to accomplish robust reprogramming of human adult cells such as fibroblasts or keratinocytes employing recombinant proteins. Optimized expression and purification protocols are needed to be established that, for example, exploit the insoluble fraction of recombinant Sox2-TAT by purification under denaturing conditions. Moreover, alternative expression hosts including eukaryotic cells might enhance the derivation of soluble native Sox2-TAT protein. Sox2 protein transduction might not only be instrumental for the derivation of factor-free iPS cells but also for the analysis of the reprogramming mechanism by providing a tool to precisely determine the duration and dose of Sox2 induction.
